# Start, stop, resume, and proceed: ZmSSRP1 mediates the progression of RNA polymerase II and kernel development in maize

**DOI:** 10.1093/plcell/koaf113

**Published:** 2025-05-07

**Authors:** Christian Damian Lorenzo

**Affiliations:** The Plant Cell, American Society of Plant Biologists; Center for Plant Systems Biology, VIB, Gent B-9052, Belgium; Department of Plant Biotechnology and Bioinformatics, Ghent University, Gent B-9052, Belgium

Gene transcription is an essential process carried out by RNA Polymerase II (RNAP II) through a cycle of 4 key steps. It begins with initiation, followed by a promoter-proximal pausing phase, during which serine residues in the carboxy-terminal domain of RNAP II become phosphorylated. Next comes elongation, where the full RNA transcript is synthesized, and finally, termination, during which the mRNA is processed and the poly(A) tail is added (Liu et al. 2015). The journey of RNAP II through a gene is anything but easy, and the elongation step, particularly, can be influenced by several factors (Obermeyer et al. 2023). Nucleosomes (formed by DNA wrapped around an octamer of histones), for example, can hinder RNAP II movement. The complex FACILITATES CHROMATIN TRANSCRIPTION (FACT), composed of the 2 subunits STRUCTURE SPECIFIC RECOGNITION PROTEIN1 (SSRP1) and SUPPRESSOR OF TY (SPT16), promotes both nucleosome disassembly and reassembly, clearing the path for RNAP II to move along (Formosa and Winston 2020). FACT has been observed to co-purify with RNAP II and has also been implicated in the regulation of other steps of transcription (Tettey et al. 2019). Although RNAP II and FACT complex genes have been well characterized in *Arabidopsis*, the conservation of their functions in other plants is under investigation.

In recent research, **Jin-Yu Wang and collaborators (Wang et al. 2025a)** identified a maize mutant, *dek59-1*, with a reduced endosperm phenotype associated with a maize ortholog of *SSRP1*. *dek59-1* was identified in an EMS screen due to its small kernels, resulting from a reduction in seed starch content. Positional cloning of *dek59-1* led to a change between the wild type and the mutant in the last nucleotide of intron 12 of *ZmSSRP1*, a region critical for proper splicing. Wang et al. found that the mutation caused 2 types of misspliced events: one with a 16-nucleotide addition leading to a loss of function, and another with a 15-nucleotide deletion that led to a fully translated protein detectable by western blot. The authors further noticed that *dek59-1* presented cell cycle–related problems, such as reduced cell number in endosperm tissue and disturbed endoreduplication values compared to the wild type. These results were supported by RNA-seq analysis between the wild type and *dek59-1*. Chromatin immunoprecipitation sequencing assays using a specific antibody against ZmSSRP1 revealed that this protein binds more to genic regions, displaying more occupancy on highly expressed genes. The group also observed through co-immunoprecipitation that ZmSSRP1 indirectly interacts with the maize RNAP II.

To investigate the nature of this interaction in vivo, the authors performed chromatin immunoprecipitation-seq assays in *dek59-1* and wild-type lines using an antibody specific for RNAP II Ser2P. The mutation of ZmSSRP1 leads to a shift of RNAP II distribution toward the transcription starting site and decreases at the 3′ end of genes, suggesting reduced polymerase processivity. Additionally, chromatin accessibility in +1 nucleosomes (a barrier for RNAP II) is highly decreased in *dek59-1* mutants as measured by ATAC-seq, hinting that ZmSSRP1 mutation could lead to reduced RNAP II progression due to less accessible chromatin. This effect was more substantial for longer genes than for shorter ones. Finally, the researchers also discovered that arrested ZmSSRP1 in *dek59-1* is eliminated through proteasome-mediated degradation (see Figure).

**Figure.**
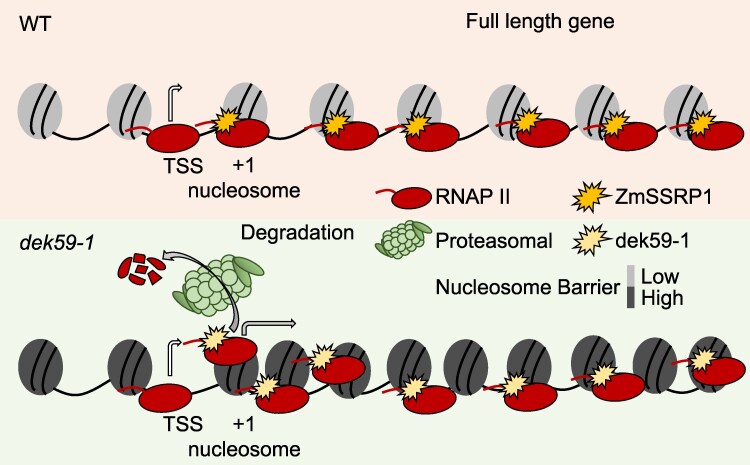
Model depicting ZmSSRP1 mechanism of action in transcript elongation in wild type and its misregulation, arrest, and subsequent degradation mediated by the proteasome in *dek59-1* mutants. Adapted from Wang et al. (2025a, 2025b), Figure 6.

Thus, Wang et al. have shed light on the conserved role of SSRP1 in transcript elongation in maize and how its misregulation can lead to severe phenotypes, opening the path to future studies of the pivotal functions of RNAP II and FACT complex members in other plant species.

## Recent related articles in *The Plant Cell*

- Liu et al. (2025) shed light on how 2 different transcription factors, *MULTI-FLORET SPIKELET1* (*TaMFS1*) and the *STRUCTURE-SPECIFIC RECOGNITION PROTEIN 1* (*TaSSRP1*), regulate VRT-A2 expression, a gene with important agronomical breeding potential in wheat.
- Wang et al. (2025b) described an abundance of alternative transcription starting sites present the in coding regions of several tissue-specific soybean genes. These alternative transcription starting sites within CDSs were epigenetically tuned and associated with tissue-specific functions.
- Sheng et al. (2025) isolated a gene *COMPACT PLANT 3* (*CT3*), whose mutation leads to a compact plant architecture and increased plant gene in high-density stands. CT3 acts as a co-regulator interacting with other transcription factors and increasing their DNA binding affinity.

## Dive Curated Terms

The following phenotypic, genotypic, and functional terms are of significance to the work described in this paper:

SPT16 Gramene: AT4G10710
SPT16 Araport: AT4G10710
